# Improving the S-Shape Solar Radiation Estimation Method for Supporting Crop Models

**DOI:** 10.1100/2012/768530

**Published:** 2012-05-03

**Authors:** Nándor Fodor

**Affiliations:** Crop Modeling research group, Department of Environmental Informatics, Institute for Soil Sciences and Agricultural Chemistry, Centre for Agricultural Research, Hungarian Academy of Sciences, Herman Ottó út 15, H-1022 Budapest, Hungary

## Abstract

In line with the critical comments formulated in relation to the S-shape global solar radiation estimation method, the original formula was improved via a 5-step procedure. The improved method was compared to four-reference methods on a large North-American database. According to the investigated error indicators, the final 7-parameter S-shape method has the same or even better estimation efficiency than the original formula. The improved formula is able to provide radiation estimates with a particularly low error pattern index (PI_doy_) which is especially important concerning the usability of the estimated radiation values in crop models. Using site-specific calibration, the radiation estimates of the improved S-shape method caused an average of 2.72 ± 1.02 (*α* = 0.05) relative error in the calculated biomass. Using only readily available site specific metadata the radiation estimates caused less than 5% relative error in the crop model calculations when they were used for locations in the middle, plain territories of the USA.

## 1. Introduction

Solar radiation is the main driving force of the processes in the atmosphere, as well as in the biosphere, including the rhizosphere. Therefore, measured daily global solar radiation is an important factor in most cropping systems and water balance models. Knowledge of solar radiation data is also indispensable for many solar-energy-related applications. High-quality pyranometers are quite expensive and the accurate measurement requires careful maintenance, including periodical calibration.

The scarcity of good quality solar radiation data series can be a major limitation to the use of crop models. To circumvent this problem, numerous radiation estimation methods were developed using commonly measured meteorological variables, such us sunshine hours, temperature, and precipitation [1], as well as [2] gave a comprehensive overview and carried out an in-depth analysis of the temperature and precipitation based global radiation models.

A good number of radiation estimation methods are based on the daily sunshine hours [3–7]. These methods can provide more accurate estimations than the temperature-based procedures [8]. Daily global radiation has a much stronger physical relationship with the sunshine hours than with the daily temperature change, not to mention that the causal relationship is reversed in the latter case. Despite of this, the simple fact that temperature and precipitation are measured at many more locations than sunshine hours provides the grounds for the temperature-based methods.

In this paper, we are focusing on the temperature and precipitation-based models. The central component of the temperature-based models is the function describing the relationship between the fraction of clear day (*F*_cd_) and the daily or the smoothed daily temperature range (Δ*T*). Certain radiation estimating methods (belonging to the Hargreaves model [9] family) use a simple power function of Δ*T* for calculating *F*_cd_, while others (belonging to the Bristow-Campbell model [10] family) compose the power function with an exponential association function. Both function types have limitations when used for describing the *F*_cd_-Δ*T* relationship. Both tend to zero as Δ*T* approaches zero, moreover, the functions of the first kind are not bounded above. These are obvious weaknesses, since the global solar radiation is greater than zero even on days when the temperature change is zero, and a function that is not bounded above may provide radiation estimates exceeding the physically possible value on days with very large temperature differences (Section 2.2). The majority of the temperature-based models just ignore (or neglect) these limitations. Models taking the effect of precipitation amount or occurrence into account do not solve this problem as it will be shown later.

In our previous study [11], an S-shape function was introduced for calculating the fraction of clear day from the daily smoothed temperature change based on analogies from soil science. This function type eliminates the problems mentioned in the previous paragraph. This paper received critical comments from [12] listing four major concerns about the new radiation estimation method and its evaluation: (1) too many parameters are used in the model, (2) the Donatelli-Campbell method is not the best competing model for comparative evaluation, (3) the analysis of model performance is inadequate, and (4) the knowledge about the global radiation temperature difference relationship is neither extended nor adequately discussed.

The present study was motivated by these four critical comments. From a scientific point of view criticism 4 is the most interesting. The authors hold that the basic concept formulated by Bristow and Campbell (1984) [10] is valid, though the relationship between solar radiation and Δ*T* is influenced by several factors which are thoroughly discussed by [13]. Although, the primarily goal of research is to extend our knowledge on the global radiation-temperature difference relationship, or rather to reveal more factors/phenomena modifying the base relationship, there is another important focus of radiation estimation model development. Realism is the main issue, but functionality is also important. It is important to pursue a better understating of the phenomenon in question but meanwhile, there are a couple of practical applications which require only better estimations and are not necessarily concerned about the physical processes in the background. It is interesting to that even theoretically incorrect (unrealistic), models can be functional and useful: Ptolemy's geocentric model could be a case in point. It was successfully used for centuries, even America was discovered by Columbus and the globe was circumnavigated by Magellan using this model for navigation.

In this study, we are focusing on the functionality as we try to enhance the S-shape method according to the following objectives: (1) decrease the number of parameters, (2) add more recent reference models for performance comparison, (3) improve the analysis of model performance, (4) investigate the usability of estimated radiation data in crop models, and (5) extend the estimations for sites without radiation measurement using only geographical data and meteorological metadata.

## 2. Materials and Methods

The same database of 109 USA meteorological stations [14] was used for the present study as for the paper introducing the S-shape method [11]. Data from the 1961–1990 period were used for each site. The average temperatures, the average annual cumulative solar radiations, and the average annual precipitation amounts of the sites range between 6.3 and 25.5°C, 4004 and 7099 MJ m^−2^, and 375 and 3359 mm, respectively. The investigated stations represent the semiarid, humid subtropical, marine west coast, and humid continental climatic regions of the United States.

### 2.1. Decreasing the Number of Parameters in the S-Shape Method

The S-shape method [11] uses the following formulae for radiation estimation:

(1) $$\begin{array}{c} R_{s}=R_{a}\cdot \;\tau \cdot F_{\text{cd}}, \end{array}$$

(2) $$\begin{array}{cc} \tau & =a+b\cdot \cos\left(i\cdot \frac{2\pi }{365}\right)+c\cdot \sin\left(i\cdot \frac{2\pi }{365}\right)\\ & \;+d\cdot \cos\left(i\cdot \frac{4\pi }{365}\right)+e\cdot \sin\left(i\cdot \frac{4\pi }{365}\right), \end{array}$$

(3) $$\begin{array}{c} F_{\text{cd}}=1-\frac{1-f}{{\left(1+{\left(g\cdot \Delta T\right)}^{n}\right)}^{m}}. \end{array}$$

**Figure 1:**
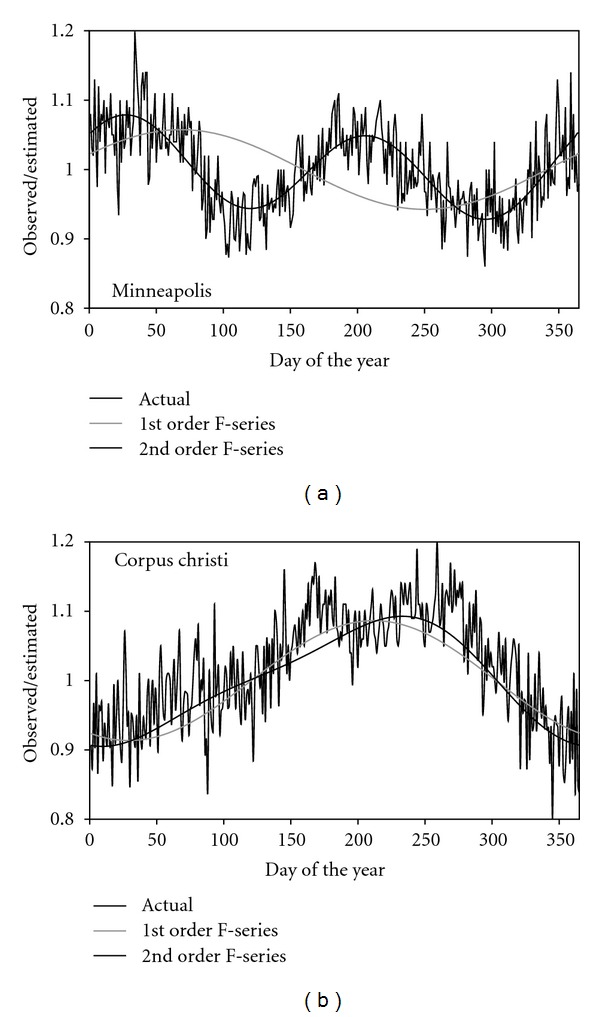
*R* ^obs^/*R* ^est^ ratios plotted against DOY and the fitted ^ 1^F- and ^ 2^F-series.

*R*_*s*_ and *R*_*a*_ are the daily global radiation and the extraterrestrial radiation, respectively. *τ* denotes the clear sky transmissivity, *i* is the day of the year, while *a*, *b*, *c*, *d*, *e*, *f*, *g*, *n*, and *m* are site-specific parameters. Equation (2) (a second order Fourier series, ^2^F-series) accounts for the annual changes of the clear sky transmissivity. Since (3) is parameterized separately for wet and dry days for each season the total number of parameters to be determined during site-specific calibration is 5 + 8 × 4 = 37. First, we wanted to decrease the number of parameters but only as long as the good model performance was assured. This plan was carried out in five steps:

- The effect of using a 1st-order Fourier series (^1^F-series) or a simple constant value instead of (2) was investigated.
- Despite of its seasonal parameterization, the residuals of the S-shape method exhibited considerable patterns when plotted against the day of the year (DOY). The PI_doy_ index of the original S-shape method is relatively high as it was already indicated in [11]. To decrease this figure, a double-step parameterization process was introduced. The idea was borrowed from [15], though it is implemented in a different way. First the parameters of (2) and (3) are determined with a nonlinear regression method, than the average values of the *R* ^obs^/*R* ^est^ ratios are calculated for each DOY. This plot exhibits a quite pronounced though, site-specific annual pattern (Figure 1) which could be approximated with a ^2^F-series or a ^1^F-series with their constant term is set to 1. Using this F-series as a multiplicative term in the radiation estimation equation, the seasonal parameterization becomes unnecessary.
- In the original S-shape method the *F*_cd_-Δ*T* relationship was formulated based on van Genuchten's soil water retention curve [16]. A similar S-shaped curve was proposed earlier by [17] having fewer parameters for describing the SWRC. According to this function, the following formula was implemented in the S-shape method:
  (4) $$\begin{array}{c} F_{\text{cd}}=1-\frac{1-f}{1+{\left(g\cdot \Delta T\right)}^{n}} \end{array}.$$
- [1] incorporated the effect of precipitation occurrence (as a multiplicative factor, see (9)) in their method though, according to their formula the parameters of the *F*_cd_(Δ*T*) function for wet and dry days are interrelated. Leaving the separate parameterization of (3) for wet and dry days, the idea of [1] was implemented in the S-shape method.
- Following the idea of [2], a correlation analysis was carried out. The constant clear sky transmissivity values of the 109 investigated sites were correlated to the geographical data (latitude, altitude) and meteorological metadata (average temperature, average diurnal temperature difference, and average annual precipitation) of the stations. A significant (*r* = 0.727***) relationship was found between the average diurnal temperature range (Δ*T*_avg_) and the clear sky transmissivity: *τ* = 0.00591 · Δ*T*_avg_ + 0.6758.

Based on I–V, the estimation performance of the following general formula was studied

(5) $$R_{s}=R_{a}\cdot \tau \cdot F_{\text{cd}}\cdot \left(1+q\cdot R\right)\cdot F_{s},$$

where *q* is a site-specific parameter, *R* denotes the precipitation occurrence (1 for wet and 0 for dry days). *F*_*s*_ stands for a ^1^F or a ^2^F-series with a constant term of unity. For the investigation of the different types of (5), the following notations are introduced (Table 1).

### 2.2. Extending the List of Reference Models for Performance Comparison

The original S-shape method [11] was compared with the Donatelli-Campbell (DC) model [18]. In the present study, the following methods are used as reference models:

**DC Model**

(6) $$\begin{array}{cc} R_{s} & =R_{a}\cdot \tau \cdot (1-\exp(-b\cdot 0.017\cdot \exp\left(\exp\left(-0.053\cdot T_{a}\right)\right)\\ & \;\;\;\;\;\cdot \exp\left(\frac{T_{\min}}{c}\right)\cdot \Delta T^{2})), \end{array}$$

where *τ*, *b* and *c* are site specific parameters. *T*_*a*_ and *T*_min⁡_ denote the daily average and minimum temperatures.

**Donatelli-Bellocchi (DB) Model [19]**

(7) $$\begin{array}{cc} R_{s} & =R_{a}\cdot \tau \\ & \;\cdot (1+b\cdot (\sin\left(i\cdot c\cdot \frac{\pi }{180}\right)\\ & \;\;\;\;\;+\cos(i\cdot \left(1-1.9\cdot \text{frac}\left(c\right)+3.83\cdot {\text{frac}}^{2}\left(c\right)\right)\\ & \;\;\;\;\;\;\;\;\;\cdot \frac{\pi }{180})))\\ & \;\cdot \left(1-\exp\left(\frac{-d\cdot \Delta T^{2}}{\Delta T_{w}}\right)\right), \end{array}$$

where *τ*, *b*, *c*, and *d* are site specific parameters and *i* is the day of the year. 0 < *c* ≤ 0.5 or 1 < *c* ≤ 1.5. Δ*T*_*w*_ denotes the moving weekly averages of the diurnal temperature differences. The frac function returns the fractional part of a real number.

**Hunt-Kuchar-Swanton (HKS) Model [20]**

(8) $$\begin{array}{c} R_{s}=R_{a}\cdot a\cdot \Delta T^{0.5}+b\cdot T_{\max}+c\cdot P+d\cdot P^{2}+e, \end{array}$$

where *a*, *b*, *c*, *d*, and *e* are site specific parameters. *T*_max⁡_ and *P* denote the daily maximum temperature and precipitation amount, respectively.

**Liu-Scott (LS) Model [1]**

(9) $$\begin{array}{cc} R_{s} & =R_{a}\cdot a\cdot \left(1-\exp\left(-b\cdot \Delta T^{c}\right)\right)\\ & \;\cdot \left(1+d\cdot R_{i-1}+e\cdot R_{i}+f\cdot R_{i+1}\right)+g, \end{array}$$

where *a*, *b*, *c*, *d*, *e*, *f*, and *g* are site-specific parameters. *R*_*i*_ denote the precipitation occurrence on the *i*th day of the year (1 for wet and 0 for dry days).

The “help” section of the RadEst software [15] suggests using the DC model for temperate areas and the DB model for tropical areas. Since the used data are limited mainly to the temperate areas, the DC model was kept in the list of the reference models. Since the HKS and the LS models take the effect of precipitation into account in different ways, both were used for performance comparison.

The model developed by [21] was also considered in the comparison, but finally the idea was abandoned for two reasons. One, this model did not outperform the above listed models in previous comparative studies (e.g., in [22]). Two, since the models in the Hargreaves model family [2] are not bounded above they could result in unrealistic radiation estimates for days with large temperature difference. We investigated the model proposed by [21] and found more than 2500 days (out of the c.c. 40000 days of the 109 stations) when the estimated radiation value was greater than the physical upper limit calculated with the formula proposed by [23]. We found almost a hundred days when the estimated sky transmissivity was greater than 0.9. The same problem was found while using the HKS model: more than 2080 days with unrealistically high estimated radiation.

It has to be noted that both the DC and the DB model have an unrealistic characteristic as their limit is zero when Δ*T* approaches zero. True enough, the proportion of days with less then 5°C temperature difference is only around 10% in the used database. The HKS and LS models share this characteristic to some extent, however, the temperature range dependence of the estimated radiation is not as explicit as for the DC and DB models. Neither [20] nor [1] discussed the problem related to the ranges of the parameters in (8) and (9). Allowing parameter *e* in (8) and parameter *g* in (9) to be less than zero during the parameterization might result in negative estimated radiation values for dry days with small temperature difference. 1490 and 17 days were found for the 109 investigated stations when the estimated radiation was less than zero calculated with the HKS and the LS models, respectively.

### 2.3. Improving the Analysis of Model Performance

In [24] three model performance indicator types were introduced: accuracy (measured with, e.g., RMSE), correlation (measured with, e.g., *R* ^2^), and pattern (measured with, e.g., distribution of residuals over the day of the year, PI_doy_). In the present study these indices are used defined by the following formulae:

(10) $$\begin{array}{c} R^{2}={\left[\frac{\sum_{i=1}^{n}\left(R_{s_{i}}^{\text{obs}}-M^{\text{obs}}\right)\cdot \left(R_{s_{i}}^{\text{est}}-M^{\text{est}}\right)}{\sqrt{\sum_{i=1}^{n}{\left(R_{s_{i}}^{\text{obs}}-M^{\text{obs}}\right)}^{2}}\cdot \sqrt{\sum_{i=1}^{n}{\left(R_{s_{i}}^{\text{est}}-M^{\text{est}}\right)}^{2}}}\right]}^{2}, \end{array}$$

(11) $$\begin{array}{c} \text{RMSE}=\sqrt{\frac{\sum_{i=1}^{n}{\left(R_{s_{i}}^{\text{obs}}-R_{s_{i}}^{\text{est}}\right)}^{2}}{n}}, \end{array}$$

(12) $$\begin{array}{cc} & {\text{PI}}_{\text{doy}}\\ & =\underset{k,m=1,2,3,4;\;k\neq m}{\max}\left|\frac{1}{n_{k}}\cdot \overset{n_{k}}{\underset{i=1}{\sum }}\left(R_{s_{i}}^{\text{est}}-R_{s_{i}}^{\text{obs}}\right)-\frac{1}{n_{m}}\cdot \overset{n_{m}}{\underset{i=1}{\sum }}\left(R_{s_{i}}^{\text{est}}-R_{s_{i}}^{\text{obs}}\right)\right| \end{array}.$$

*R* ^est^ and *R* ^obs^ are the estimated and the observed radiation values, *n* denotes the number of data pairs. For calculating PI_doy_ the year was distributed for four intervals including *n*_*k*=1–4_ days as it was proposed by [25]. The PI_*T*min⁡_ indicator was also calculated for each model using (12). In this case, the range of the observed *T*_min⁡_ values was distributed for equal intervals and the days of the data series were grouped into these intervals. Though, Bellocchi et al. [24] developed a fuzzy-based metrics integrating the previously mentioned error indicator types, all the indices above will be presented throughout the study to show all aspects of the model performance analysis.

### 2.4. Usability of Estimated Radiation Data in Crop Models

The 4M crop simulation model [26, 27] has been used in the study. 4M is a CERES [28] clone based on the source code of the CERES model [29]. Corn production estimations were calculated for the 109 sites with 4M using measured and estimated radiation of the 1961–1990 period. Estimated radiation data were obtained by using the DC, DB, HKS, LS, and the S-shape (0-2-1-4) methods. Data of a loamy sample soil profile as well as of crop-specific parameters of a FAO 400 corn cultivar from the DSSAT ver. 3.5 software package [30] were used in the simulations. The sowing and harvest dates as well as the sowing depth and plant population were set to April 25, September 25, 6 cm and 7 plant m^−2^, respectively. Though, it is obvious that the above settings are not optimal ones for many of the investigated sites, for the sake of simplicity, these were used for all of the 109 locations.

Nutrient stresses were switched off during the simulations. Cumulative evapotranspiration, yield as well as biomass outputs obtained with measured and estimated radiation were compared using *R* ^2^, RMSE, the mean relative error (MRE), and paired *t*-test results. A study of [31] on the sensitivity of crop models to the inaccuracies of meteorological observations showed that the uncertainty caused by the systematic errors of the measured global radiation can be up to 10% relative error for the calculated yield. This threshold (acceptance limit) was used for deciding whether the radiation estimation is acceptable for the crop model or not. If the difference between the model results obtained by using estimated radiation and the ones obtained by using measured radiation is less than 10%, the radiation estimation was said to be acceptable.

### 2.5. Extend the Estimations for Sites without Radiation Measurement

**Figure 2:**
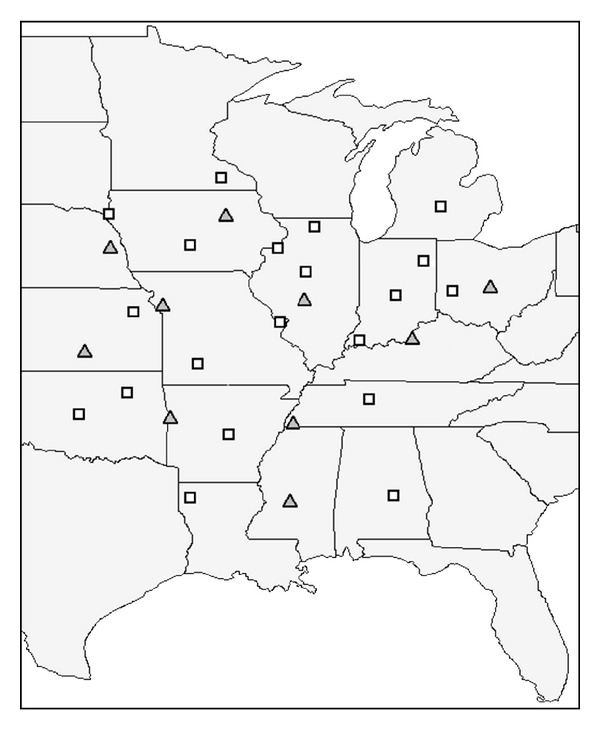
Location of the 20 and 10 stations in the central territory of the USA whose data were used for calibrating and validating the S-shape method. Squares and triangles denote the calibration and validation sites, respectively.

Further simplification of the 0-2-1-4 version (Table 1) of the S-shape method, (13) was investigated for a subset of the database of the 109 stations covering an area of about 1,000,000 km^2^ in the central part of the US mainland between the Rocky Mountains and the Appalachian Mountains (Figure 2).

(13) $$\begin{array}{c} R_{s}=R_{a}\cdot \tau \cdot \left(1-\frac{1-a}{1+{\left(b\cdot \Delta T\right)}^{2.285}}\right)\cdot \left(1+c\cdot R\right)\cdot F_{s} \end{array},$$

(14) $$\begin{array}{c} \tau =0.00591\cdot \Delta T_{\text{avg}}+0.6758 \end{array},$$

(15) $$\begin{array}{cc} F_{s} & =1+d\cdot \cos\left(i\cdot \frac{2\pi }{365}\right)+e\cdot \sin\left(i\cdot \frac{2\pi }{365}\right)\\ & \;+f\cdot \cos\left(i\cdot \frac{4\pi }{365}\right)+g\cdot \sin\left(i\cdot \frac{4\pi }{365}\right). \end{array}$$

Data of 20 stations were used for model calibration (Figure 2). The parameters *a*–*g* in (13) were determined by site specific parameterization. Since the coefficient of variation (CV) of parameters *a*, *b*, *c*, and *d* was relatively low (ranging between 6.7 and 16.9%), these parameters were approximated by their simple means. The rest of the parameters (CV = 28.5 − 104.7%) were correlated to the geographical data (latitude, altitude) and meteorological metadata (average temperature, average diurnal temperature difference, and average annual precipitation) of the stations as it was proposed by [2]. The 0-2-1-4 version of the S-shape method calibrated with the previously introduced procedure was then validated using the data of 10 stations (Figure 1). The performance of this version of the S-shape method (having zero parameters to be determined by site-specific parameterization) was compared to those of the DC, DB, HKS, and LS models using the introduced error indicators. The estimated radiation values were used also as crop model inputs and the obtained biomass results were compared to those obtained with measured radiation for the 10 validation sites.

## 3. Results and Discussion

### 3.1. Analysis of the Different Types of the S-Shape Method

**Figure 3:**
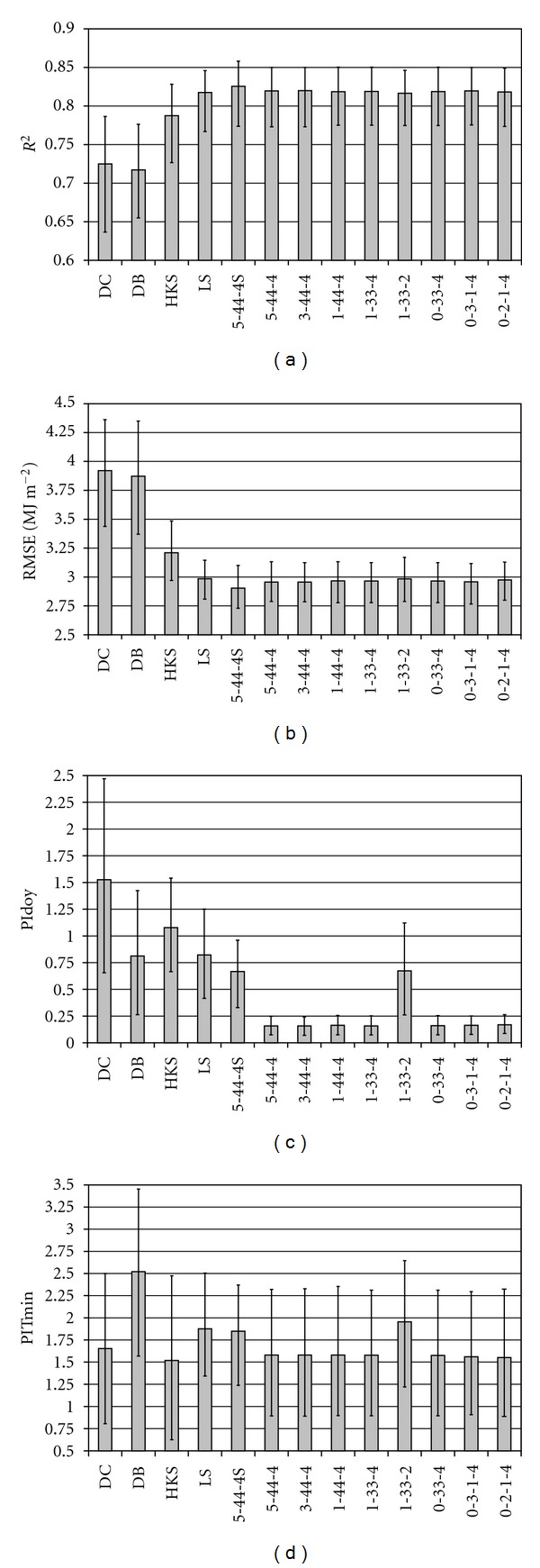
Error indices of the investigated radiation estimation methods. See the explanations of the different S-shape methods in Table 1. The bars show the average values for the 109 stations. Ticks on the bars represent the 10% and 90% percentiles.

According to Figure 3, the parameter number of the original S-shape method (5-44-4S) could be decreased considerably without weakening the model performance. Its general formula, (5), is not sensitive to the form of the clear sky transmissivity term, whether ^2^F- or ^1^F-series or a constant value is used (compare 5-44-4, 3-44-4 and 1-44-4 in Figure 3). Or, more likely, the Fourier series used for eliminating the seasonal trends form the residuals (*F*_*s*_ in (5)) may compensate the effects of using simpler *τ* terms. The double-step parameterization process (introducing *F*_*s*_ in (5)) was the most successful step. This made it possible to abandon the seasonal parameterization decreasing the number of parameters from 37 to 17 while it successfully filters out the seasonal trends from the annual course of the residuals resulting in considerably smaller PI_doy_ indices (compare 5-44-S4 and 5-44-4 in Figure 3). As it was demonstrated on Figure 1(a) ^1^F-series is not flexible enough to describe the pattern of bias for many sites (compare 1-33-4 and 1-33-2 in Figure 3). Using a simpler S-shaped curve for describing the *F*_cd_-Δ*T* relationship (4) did not decrease the model performance (compare 1-44-4 and 1-33-4 in Figure 3). Setting parameter *n* to an average value (*n* = 2.285) for all of the investigated sites did not affect the model performance (see 0-2-1-4 in Figure 3). When constant values were used for the parameters *f* and *g* in (4) the PI_doy_ index increased considerably, over 0.3. Using a parameter estimation equation for calculating the clear sky transmissivity (*τ* = 0.00591 · Δ*T*_avg_ + 0.6758) and taking the effect of precipitation occurrence into account with a multiplicative term (1 + *q* · *R* in (5)) did not alter the model performance (compare 1-33-4, 0-33-4 and 0-3-1-4 in Figure 3). The result is a 7-parameter formula that has slightly worse accuracy and correlation indices but considerably better Pattern indices than that of the original, 37-parameter S-shape method.

The final, 7-parameter formula (S^0-2-1-4^) performed better than the reference models according to the error indices (Figure 3). The only exception was the HKS model which had a slightly smaller PI_*T*min⁡_ index than that of the S-shape method. Note that the regression equation of [20] uses the daily maximum temperature which is in close relationship with *T*_min⁡⁡_ (*r* > 0.9 according to the used database). This fact probably explains the well performance of the HKS method as far as the PI_*T*min⁡_ index is concerned.

Including information about precipitation (occurrence or amount) considerably improves model accuracy (compare DC and DB with HKS and LS in Figure 3). Considering the Pattern indices (PI_doy_ and PI_*T*min⁡_) the temperature-based models (DC and DB) can outperform the temperature and precipitation-based models (HKS and LS). This is because of their parameterization process during which parameter *c* in (6) and parameters *b* and *c* in (7) are determined so that PI_doy_ and PI_*T*min⁡  _would be minimal for the DC and DB models, respectively. Despite this fact, the S^0-2-1-4^ method was able to result smaller pattern indices than that of the DC and DB models. The S^0-2-1-4^ model is especially advantageous regarding its particularly low PI_doy_ index owing to the ^2^F-series in (5).

### 3.2. Usability of Estimated Radiation Data in Crop Models

**Figure 4:**
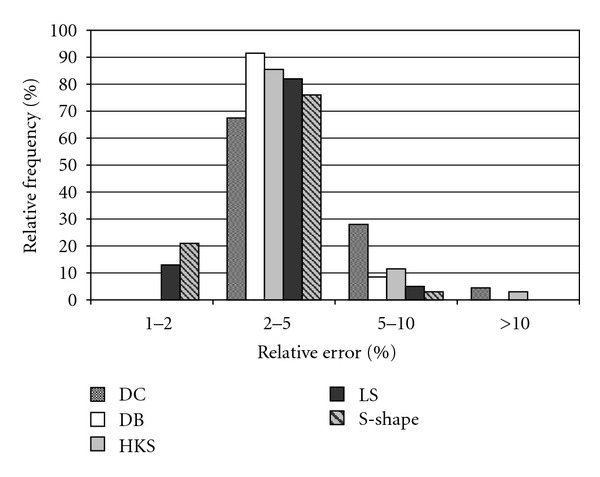
Distribution of the relative errors of biomass simulations. The crop model biomass outputs obtained with observed and estimated radiation data were compared. Radiation estimations of 5 methods (DC, DB, HKS, LS and S-shape) were used for crop model simulation.

Though, the annual cumulative evapotranspirations as well as the yields were also investigated only the biomass results (Table 2 and Figure 4) are presented here since the results were very similar for the three output variables. Despite their relatively moderate radiation estimation performance the estimations of the temperature-based methods were usable for the crop models meaning that they did not cause extreme errors in the crop model results. It is especially true for the DB method whose estimations did not cause >10% relative errors for either the stations or the years. This result highlights the importance of the PI_doy_ index when the usability of the radiation estimates in crop models is concerned. Though the HKS methods had considerably better correlation and accuracy indices (Figure 3) than the DB method, which had better PI_doy_ index, the estimations of the latter method turned out to be more usable for the crop model (resulting smaller errors) than that of the HKS method. Crop models are more sensitive to consistent errors (bias) of radiation than to random errors. The estimated radiation could be more on a day and then less on another than the observed radiation but still, the biomass calculated with estimated radiation is similar to that calculated with observed radiation at the end, resulting small error in the crop model outputs. But if the estimation is biased the calculated biomass will also be biased resulting in greater crop model output errors. The small PI_doy_ index indicates moderate seasonal trends in the residuals meaning random estimation errors across the year which is favorable for crop model applications.

**Figure 5:**
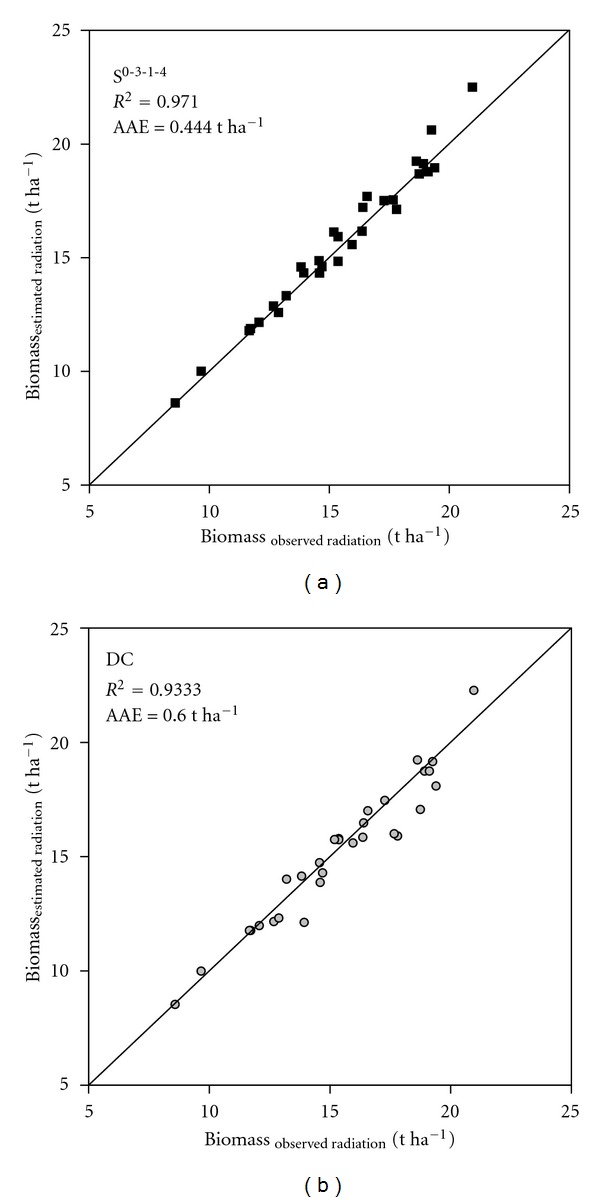
Biomass results obtained with observed and estimated (the S^0-2-1-4^ and the DC methods were used) radiation. Tulsa, OK, 1961–1990. AAE denotes the average absolute error.

The DB, LS, and the S^0-2-1-4^ methods did not produce >10% relative errors in the biomass calculations. Their radiation estimates were acceptable for all of the stations and for all of the years. In 96.7% of the cases the estimated radiation data obtained with the S^0-2-1-4^ method caused <5% relative errors. The results of the *t*-tests were found misleading when they were used for evaluating the crop model results. This problem is demonstrated via the example of the biomass results obtained for Tulsa, OK. The *t*-test showed a significant difference between the biomass values calculated by using the observed and the estimated radiation obtained with the S^0-2-1-4^ method despite the fact that the PI_doy_ index of the radiation estimation method was only 0.066 for this site. Interestingly, the *t*-test showed no significant difference for the same site for the DC method which had PI_doy_ = 0.442. The averages of the radiation estimation-based biomass calculation errors were practically identical for the two methods: 218 and −217 kg ha^−1^ for the S^0-2-1-4^ and the DC methods, respectively. Since the standard deviation of the errors were considerably greater for the DC method (799 kg ha^−1^) than that of the S^0-2-1-4^ method (553 kg ha^−1^), the *t*-test resulted in significantly smaller *T* value for the DC method despite the fact that the S^0-2-1-4^ method performed better according to the crop model results (Figure 5).

### 3.3. Extend the Estimations for Sites without Radiation Measurement

The following parameter values and parameter estimation equations were obtained for the parameters of (13) based on the 20 validation sites (Figure 2).

(16) $$\begin{array}{c} \tau =0.00591\cdot \Delta T_{\text{avg}}+0.6758,\\ a=0.476;\;b=0.106;\;c=2.25;\;d=-0.259,\\ e=-0.00377\cdot \Delta T_{\text{avg}}+0.0312\;\left(r=-0.816^{***}\right),\\ f=-0.00341\cdot \Delta T_{\text{avg}}+0.0597\;\left(r=-0.815^{***}\right),\\ g=-0.00076\cdot \Delta T_{\text{avg}}+0.0320\;\left(r=-0.400^{***}\right),\\ h=-0.0036\cdot \Delta T_{\text{avg}}+0.0755\;\left(r=-0.905^{***}\right), \end{array}$$

where Δ*T*_avg_ denotes the average diurnal temperature difference of the site. Correlation coefficients are shown in the brackets where the significance of the correlation is also indicated. The performance of the S^0-2-1-4^ as well as the reference methods is presented in Table 3.

Despite the fact that the reference methods were parameterized for each site the S^0-2-1-4^ method (using only Δ*T*_avg_  as site specific metadata) performed well in the comparison. Regarding the correlation and the accuracy indices the S^0-2-1-4^ method had better figures than those of the DC, DB, and HKS methods for all of the sites (Table 3). With the exception of two locations the same was true for the PI_doy_ index. Though the LS method proved to be more accurate (smaller RMSE) in the comparison, the S^0-2-1-4^ method explicitly outperformed it as far as the PI_doy_ index was concerned. The radiation estimates of the S^0-3-1-4^ method were usable for the crop model for all of the ten validation sites causing only 3.3 ± 0.7% (*α* = 0.05) relative error in average in the calculated biomass (Table 4).

## 4. Conclusions

The S-shape global solar radiation estimation method, originally formulated using analogies from soil science, has been improved via a 5-step procedure. The improved method was tested on a large North-American database along with four reference methods. The new formula has considerably fewer parameters than the original one while its performance indicators are practically the same or better. The final 7-parameter S-shape method was the best performing model among the investigated procedures based on the average error indicators. The most favorable characteristic of the improved formula is that it is able to provide radiation estimates with considerably lower PI_doy_ pattern index than other estimation methods. This characteristic is especially important concerning the usability of the estimated radiation values in crop models.

Despite of their radiation estimation performances estimates of all of the investigated methods were found to be usable in crop models causing acceptably small errors in the model calculations. The radiation estimates of the improved S-shape method caused an average of 2.72 ± 1.02  (*α* = 0.05) relative error in the calculated biomass. Using only readily available site specific metadata (Δ*T*_avg_) the estimations of the improved S-shape method were successfully extended for sites without radiation measurement. According to the validation results, the radiation estimates cause less than 5% relative error in the crop model calculations when they are used for locations in the middle, plain territories of the USA.

Based on the comparison of the estimation methods it is obvious that, if possible, the precipitation occurrence and/or precipitation amount data should be included in the radiation estimation procedure in order to obtain better estimates. It seems that the calculations of the Hargreaves model family methods (e.g., the investigated HKS method) should be limited from above by using the equation of [23] for instance, in order to avoid unrealistic radiation estimates.

The improved S-shape method could be a reliable alternative to sunshine duration-based radiation estimating procedures when only air temperature and precipitation data are available for a location in the semiarid, humid subtropical, marine west coast, and humid continental climatic regions of the United States.

## Figures and Tables

**Table 1:** Investigated types of the S-shape radiation estimation method. *Two other forms (when parameter *f* and *g* were considered to be constant) of the 0-2-1-4 type were also investigated.

Notation	Term in (5)				Number of parameters	Note
	*τ*	*F*_cd_	1 + *q* · *R*	*F*_*s*_		
5-44-4S	^2^F-series	(3)	No	No	37	S-shape as proposed in [11].
5-44-4	^2^F-series	(3)	No	^2^F-series	17	Separate parameterization for wet and dry days.
3-44-4	^1^F-series	(3)	No	^2^F-series	15	
1-44-4	constant	(3)	No	^2^F-series	13	
1-33-4	constant	(4)	No	^2^F-series	11	
1-33-2	constant	(4)	No	^1^F-series	9	
0-33-4	Estimated from Δ*T*_avg_	(4)	No	^2^F-series	10	
0-3-1-4	Estimated from Δ*T*_avg_	(4)	Yes	^2^F-series	8	
0-2-1-4*	Estimated from Δ*T*_avg_	(4)	Yes	^2^F-series	7	Parameter *n* in (4) is set to 2.285 which is the average of the *n* parameters of the 0-3-1-4 method obtained with site specific parameterization for the 109 stations.

**Table 2:** Comparison of crop model biomass outputs obtained with measured and estimated radiation. Radiation data were produced with the indicated methods. The averages and the confidence intervals (*α* = 0.05) of the error indicators were calculated based on the model results obtained for the 109 investigated stations. The *t*-tests were carried out for each station separately using 30-year long simulations.

Estimation method	*R* ^2^	RMSE (kg ha^−1^)	MRE (%)	Proportion of significant differences based on *t*-test (*α* = 0.05) (%)
DC	0.872 ± 0.033	1090 ± 105	5.31 ± 5.49	69
DB	0.937 ± 0.015	764 ± 32	3.46 ± 1.34	33
HKS	0.916 ± 0.026	845 ± 57	3.93 ± 2.55	38
LS	0.952 ± 0.012	625 ± 27	2.84 ± 1.16	15
S^0-2-1-4^	0.952 ± 0.014	599 ± 24	2.72 ± 1.02	15

**Table 3:** Comparison of the radiation estimates obtained with the indicated methods for the validation sites (Figure 2). Parameters of S^0-2-1-4^ were set according to Section 3.3. The DC, DB, HKS, and LS methods were parameterized for each site separately.

Estimation method	Error index	Columbus, OH	Fort Smith, AR	Jackson, MS	Kansas City, MO	Louisville, KY	Memphis, TN	Omaha, NE	Springfield, IL	Waterloo, IA	Wichita, KS	Average
	*R* ^2^	0.723	0.757	0.708	0.773	0.734	0.708	0.760	0.748	0.763	0.777	0.745
DC	RMSE	4.01	3.58	3.69	3.76	3.83	3.94	4.00	4.02	4.02	3.65	3.85
	PI_doy_	0.951	0.946	2.270	0.755	1.210	2.090	1.040	1.210	2.070	0.329	1.287

	*R* ^2^	0.698	0.759	0.731	0.743	0.722	0.714	0.734	0.715	0.734	0.758	0.731
DB	RMSE	4.10	3.53	3.57	3.92	3.92	3.99	4.11	4.16	3.99	3.72	3.90
	PI_doy_	0.779	0.581	0.316	0.881	0.585	0.609	0.891	0.953	1.070	0.688	0.735

	*R* ^2^	0.802	0.813	0.792	0.806	0.804	0.791	0.792	0.801	0.816	0.814	0.803
HKS	RMSE	3.21	3.04	3.08	3.32	3.21	3.30	3.55	3.35	3.19	3.17	3.24
	PI_doy_	0.986	0.740	0.972	0.789	1.010	1.090	0.965	0.894	0.973	0.677	0.910

	*R* ^2^	0.829	0.831	0.812	0.838	0.834	0.823	0.816	0.832	0.836	0.835	0.829
LS	RMSE	2.98	2.90	2.95	3.03	2.96	3.05	3.33	3.08	3.01	2.99	3.03
	PI_doy_	0.465	0.608	0.997	0.534	0.689	0.888	0.824	0.621	0.975	0.413	0.701

	*R* ^2^	0.831	0.828	0.805	0.839	0.836	0.825	0.816	0.836	0.840	0.832	0.829
S^0-2-1-4^	RMSE	3.03	2.94	3.01	3.07	2.94	3.22	3.35	3.06	2.98	3.03	3.06
	PI_doy_	0.612	0.585	0.253	0.521	0.210	0.971	0.397	0.149	0.198	0.329	0.423

**Table 4:** Error indices of the crop model biomass results for the validation sites. Asterisks denote significant differences (*α* = 0.05). Crop model results obtained with measured radiation were compared to those obtained with the S^0-2-1-4^ method parameterized according to Section 3.3.

Site	*R* ^2^	RMSE (kg ha^−1^)	MRE (%)
Columbus, OH	0.955	1222	4.6*
Fort Smith, AR	0.934	901	3.7
Jackson, MS	0.953	916	4.6*
Kansas City, MO	0.947	783	2.9
Louisville, KY	0.979	534	2.4
Omaha, NE	0.910	841	3.0
Memphis, TN	0.913	1050	5.4*
Springfield, IL	0.984	516	2.5
Waterloo, IA	0.987	533	1.9
Wichita, KS	0.988	468	2.3
